# Primary care video consulting: a view from Northern Europe

**DOI:** 10.3399/bjgp25X742641

**Published:** 2024-05-30

**Authors:** Rebecca Payne, Ulrik Bak Kirk, Veronica Milos Nymberg, Nathalie Sandal, Linda Huibers, Elisabeth Assing Hvidt

**Affiliations:** North Wales Medical School, Bangor University, Bangor, UK; DPhil student, Nuffield Department of Primary Healthcare Sciences, University of Oxford, Oxford, UK.; Department of Public Health, Aarhus University, Aarhus, Denmark; Chief Consultant, Research Unit for General Practice, Aarhus University, Aarhus, Denmark.; Center for Primary Health Care Research, Department of Clinical Sciences, Malmö, Lund University, Malmö, Sweden; University Clinic Primary Care Skåne, Region Skåne, Sweden.; National Centre for Emergency Primary Health Care, NORCE, Norwegian Research Centre, Bergen, Norway; Norwegian Air Ambulance Foundation, Oslo, Norway; Department of Global Public Health and Primary Care, University of Bergen, Bergen, Norway.; Department of Public Health, Aarhus University, Aarhus, Denmark; Senior Researcher, Research Unit for General Practice, Aarhus University, Aarhus, Denmark.; Department of Public Health, Research Unit of General Practice, University of Southern Denmark, Denmark.

## Introduction

Prior to the COVID-19 pandemic, the use of video consultation was largely confined to enthusiasts and the private sector. Private providers offered fee-per-consultation in primary care in the UK and Sweden, and had even taken on a small number of English^1^ and Swedish general practices.^2^ The Scottish health system was an outlier as it attempted to meet the needs of remote communities with a national video consulting programme.^3^ Elsewhere in Northern Europe, systematic integration within health services was rare. This changed with the onset of the COVID-19 pandemic, when video was seen as a way of augmenting the limited information received on telephone consultations and providing safer remote care. Across a range of nations, video consulting was rapidly adopted, but, as lockdowns eased and health services returned to a new normal, video usage declined whereas telephone use remained high.^4^

So where do things stand in 2025? Five years on from the start of the pandemic, primary care video consulting has not been altogether abandoned. The Danish government has now mandated that it is offered by all general practices, Sweden has integrated it within most surgeries and the national health architecture, and in Norway and Scotland it has found a niche in urgent care services.

This article examines the future role for primary care video consultation by examining use patterns in four Northern European countries.

## The Northern European picture

### Scotland

Scotland introduced video consultation across a range of specialties including general practice prior to the COVID-19 pandemic. This experience allowed rapid upscaling as the pandemic hit, with wide initial uptake across primary care. However, as restrictions eased, most practices abandoned its routine use, while a small minority incorporated it into ‘business as usual’ and continued to provide a significant proportion of care via video.

A ‘deep dive’ into these high-use practices showed that, in these practices, receptionists actively promoted the use of video when booking appointments, and that patients had a range of options for consultation, including video or telephone. All rooms, including reception, were set up for video consultation. These practices used video successfully for a wide range of conditions including paediatrics, mental health, older complex patients, and long-term condition management.^5^,^6^ This is in contrast to most other UK practices, where GPs felt that video offered little advantage over a telephone call (but brought significantly extra hassle for both doctor and patient) and was an inferior option compared to an in-person consultation.^7^

Video has also found a niche in many Scottish GP out-of-hours services, where it is often used in the initial assessment of patients, supporting decisions about which patients need to travel for further assessment, and which can be safely treated at home. An example of this use is given in Box 1. The platform has been further configured for this purpose, with a ‘consult now’ feature allowing a telephone call to be rapidly converted to video. GPs and their teams may also be involved in supporting video consultations delivered by hospital specialists to remote (often island) communities. Patients often attend such appointments from the GP surgery, supported by an administrative or clinical member of the practice team.^3^

Box 1 **Clinician’s experiences of using video consultation**

Sophie is an out-of-hours GP in a remote part of Scotland. Patients often need to travel over an hour to an in-person appointment. She has the national video consulting platform ’Near me’ page saved in her favourites list on the out-of-hours laptop. When a request comes for an appointment or home visit for a distant patient, she first telephones the patient and then converts to a video call by sending the patient a link via text. This allows her to decide with the patient as to whether they still need in-person care or can be treated remotely.

Box 2 **Patient’s experiences of using video consulting**

Jack, a 27-year-old living in Denmark, manages chronic depression and anxiety. To monitor his progress and medication dosages, he regularly consults his GP via video. Accessing these consultations is simple — he logs on to the secure My Doctor app, which enables seamless virtual check-ins. Jack values the continuity of care that video consultations provide, allowing him to speak with his regular GP. This familiarity is particularly reassuring when discussing sensitive aspects of his mental health. However, he acknowledges a drawback: the absence of physical presence can weaken therapeutic rapport and reduce the sense of human connection. While Jack finds video consultations convenient and helpful for his ongoing care, he is grateful that his treatment did not start this way. He stresses that video consultations may not be suitable for everyone, especially those in more vulnerable mental health situations who might require in-person support.

### Norway

Norway has seen a similar pattern of use develop. A lack of primary care reimbursement or financial incentives prior to the pandemic meant use of pre-pandemic video consultations in general practice were minimal.^8^ For GPs, the installation of video equipment involved additional costs, and the fee-system was explicitly restricted for reimbursement for video consultations.

The pandemic, however, prompted the rapid implementation of video consultations, supported by national guidelines and modified reimbursement schemes.^9^,^10^ While video consultations were important during the initial phases of the pandemic, the use has since stabilised to a level lower than during the peak but remain as an important option for specific types of consultations.^9^,^11^ GPs found video consultations particularly suitable for follow-up with known patients and ongoing issues, enhancing both quality and safety in comparison with telephone consultations.^9^,^12^ Financial incentives, such as coverage through the social security system, have also been essential for widespread adoption of video consultations in general practice.^8^,^10^,^12^

In the Norwegian out-of-hours services, video tools are well integrated. The communication centres operate every hour of every day of the year, with medical operators available to Norway’s 5.5 million residents. Operators in the communication centres do initial assessments of unknown callers by triaging, prioritising, advising, and initiating action. The option to use video in this setting is perceived as useful, as it allows operators to gather more situational information, which implicitly could lead to a better basis for decision making.^13^ Video is selectively used in the out-of-hours communication centres, primarily in unclear situations, visible medical issues, and for assessing children.^14^,^15^ Callers generally perceived video positively, as it provided reassurance and enhanced communication, though some experiences increased stress or discomfort related to filming an ill or injured person.^16^

### Sweden

In contrast, Sweden entered the pandemic era with video consultations used across the country, mainly through private providers of digital care. This raised concerns regarding national cost, accessibility, inequity of care, continuity of care, and effective resource utilisation. While older patients expressed ambivalence towards use of digital care, middle-aged patients in full-time employment seem to be long-term adopters of video consultations.^17^ Although Sweden did not lock down during the pandemic, individuals adjusted their help-seeking behaviours, and the percentage of remote consultations in primary care increased.^18^ The COVID-19 pandemic also acted as a catalyst for regions to integrate video consultation platforms within regular electronic healthcare systems, as part of efforts to increase accessibility and digitally transform health care.^19^

Today, video consultations are available in most Swedish regions in primary healthcare centres, along with chat-based digital tools and a national digital contact platform called 1177. se. Many private providers of digital care have bought previously established primary care centres and offer digital– physical care. New ones have emerged, offering exclusively video consultations to patients with well-defined conditions, such as obesity, high blood pressure, or acne. It remains to be proven if the implementation of video consultations might have increased accessibility to Swedish primary care. Patients who prefer digital visits are younger, healthier, and more digitally competent. Primary care staff seem to appreciate the flexibility of working in a digital context, but few consider this a full-time career option.^20^

### Denmark

Prior to the COVID-19 pandemic, video consultations had only been used on a trial basis by a few GPs in Denmark, because of the lack of financial incentives and secure technical solutions.^4^ During the first lockdown, GPs relied on existing private teleconferencing solutions for video consultations, but from April 2020 video consultations became available through the My Doctor app, which was endorsed by the Organisation of General Practitioners in Denmark. Patients who had a video consultation more frequently tended to be younger, have higher levels of education, be employed, and reside in urban areas.^21^ Box 2 provides a fictionalised example of such a patient

The use of video consultations was most prevalent during the initial phase of the COVID-19 pandemic but declined significantly over time, accounting in 2024 for only about 1.4% of all clinic consultations. During the early-stage pandemic, 1200 out of 1677 Danish GP practices (72%) used video, which decreased to 30% in the later stage. This decrease in video consultation use among GPs aligns with qualitative research findings showing that many GPs viewed video consultations primarily as a pandemic ‘crisis tool’. Thus, they saw little value in continuing to use video consultations once the immediate crisis had passed.^22^ However, as of 2025, the collective agreement for GPs mandates that all GPs offer video consultations to their patients, meaning that not offering access to video consultations is no longer an option. The growth of private providers of digital care has expanded rapidly in recent years, providing access to an online doctor via a subscription service.

In the Danish out-of-hours setting, video is used regularly in telephone triage contacts as a triage tool (about 11%). Research suggests that video contacts could reduce the frequency of referrals to a clinical consultation, home visits, or follow-up contacts,^23^ as well as add value in communication and the decision-making process for triage GPs.^24^

## What is the value of video consultation in **t**he post-pandemic era?

It is known that video carries several advantages over telephone alone.^25^ It increases rapport between doctor and patient, and allows additional clinical and contextual information to be gained through visual cues. When substituting for an in-person consultation, it can be convenient for patients, reducing travel times and increasing access. However, it requires technical competency from patients and doctors, data, and equipment,^7^ and can introduce a new dynamic to consultations, where the technology itself becomes a third, potentially disrupting, party within the consultation.^26^ Across Northern Europe, many GPs find that the extra information to be gained is displaced by the extra work involved, and have chosen to exclude the use of video, just using telephone or in-person consultations instead.^7^

Whether video consultation is appropriate can vary by patient, condition, and context. Studies indicate that GPs find video consultations particularly suitable for follow-up with known patients and ongoing issues, enhancing both quality and safety in comparison with telephone consultations. Video consultation is particularly helpful in the out-of-hours setting, where it assists triage, ensuring that the patient is directed to the right service first time, and justifies asking the patient to travel.^27^ Video consultations offer flexibility for tasks such as renewing sick-leave.^8^ As with other forms of remote consultation, video consultation is more efficient in the context of an ongoing relationship between doctor and patient.^28^ When video is used to link a patient from their local surgery to a specialist elsewhere, with examinations facilitated by the GP, it can reduce the need for travel for specialist care.^3^

Patient satisfaction with video consultations is reportedly high, and it is particularly valued by chronically ill patients with established GP relationships who require frequent follow-ups.^12^ Satisfaction is contingent on technology literacy and trust in the GP.^29^ A qualitative study on why Danish patients choose to book video consultations shows that, while video consultations provide patients with a sense of more control with their time and the outcome of the consultation, it also provides them with a sense of diminished social and emotional presence in the doctor– patient relationship. As a result, they choose the consultation type that is most likely to fit their specific situated needs (for example, efficiency or presence).^29^,^30^

## Unanswered questions regarding the future **o**f video consultation in Northern Europe

While the evidence base is rapidly growing, there remains a research gap. We know that introducing telephone consultations and e-consultations can introduce new inefficiencies and result in double handling of patients,^31^,^32^ but it is unclear whether video can reduce or exacerbate this problem.^31^ Adding video as a consultation modality risks further increasing health inequity because of inbuilt assumptions about users excluding those less able to access and effectively use digital platforms.^33^ This can be a particular risk where insufficient attention has been paid to ‘digital facilitation’ — supporting patients to use technology.^34^

Concerns have been raised that phones and webcams may apply filters on video calls, enhancing physical appearance, making unwell patients appear healthier, and leading to clinical signs being missed.^35^ Urgent research is needed to collect evidence on the use of video and its impact and risks. Clear, evidence-based guidelines should be developed and used. Across Northern Europe, most GPs still prefer to consult with patients in-person or by telephone. There remains uncertainty as to what the ‘appetite’ for video consultation will be from patients and the profession going forward. Even in Denmark, where providing a video consultation option is now mandatory, this arose from a political push rather than from patient or practitioner pull. The Swedish and Danish experiences will provide useful learning for countries still considering the future role of video in general practice.
